# Comparative Study between Pars Plana Vitrectomy with Internal Limiting Membrane Peel and Pars Plana Vitrectomy with Internal Limiting Membrane Flap Technique for Management of Traumatic Full Thickness Macular Holes

**DOI:** 10.1155/2019/1959082

**Published:** 2019-04-21

**Authors:** Hammouda Hamdy Ghoraba, Mahmoud Leila, Hashem Ghoraba, Mohamed Amin Heikal, Emad Eldin Mohamed Elgemai

**Affiliations:** ^1^Professor of Ophthalmology, Faculty of Medicine, Tanta University, Tanta, Egypt; ^2^Medical Director, Magrabi Eye Hospital, Tanta, Egypt; ^3^Associate Professor of Ophthalmology, Retina Department, Research Institute of Ophthalmology, Giza, Egypt; ^4^Ophthalmology Specialist, Magrabi Eye Hospital, Tanta, Egypt; ^5^Assistant Professor of Ophthalmology, Faculty of Medicine, Benha University, Benha, Egypt; ^6^Ophthalmology Consultant, Damanhour Teaching Hospital, Damanhour, Egypt; ^7^Ophthalmology Consultant, Magrabi Eye Hospital, Tanta, Egypt

## Abstract

**Purpose:**

To compare the efficacy of PPV and ILM peel versus PPV and IFT in patients with traumatic FTMH.

**Methods:**

Retrospective interventional comparative case series including two groups of patients with traumatic FTMH. Patients were divided into group I (ILM peel) and group II (IFT). The main outcome measure was closure of the macular hole and restoration of the foveal microstructure. The independent-samples *T*-test and ANOVA test were used to study the mean between 2 groups and calculate the *P* value, whereas the bivariate correlation procedure studied the interaction between the variables tested.

**Results:**

Group I included 28 patients. Mean preoperative MLD was 757 *µ*m. Mean preoperative BCVA was approximately 20/320. Group II included 12 patients. Mean preoperative MLD was 529.5 *µ*m. Mean preoperative BCVA was 20/320. Group I had a macular hole closure rate of 75% versus 92% in group II *P*=0.05. Mean BCVA improvement was 2.5 lines in group I versus 5 lines in group II *P*=0.02. Disrupted ELM and IS/OS was the most salient finding in both groups.

**Conclusion:**

IFT has a significantly superior anatomic and functional outcome compared to ILM peel in traumatic FTMH.

## 1. Introduction

The key success of pars plana vitrectomy (PPV) and gas tamponade for FTMH introduced by Kelly and Wendel [1, 2] was attributed to counteracting the anteroposterior vitreous traction at the perifoveal area. On the contrary, the fluid-gas exchange flattened the subretinal fluid cuff surrounding the hole, and the gas bubble then prevented fluid currents from interfering with the healing process [3–7]. The introduction of internal limiting membrane (ILM) maculorhexis was a significant rider to the original technique that greatly spurred the success rates of surgery, although with better response in smaller size holes compared to larger size holes > 400 *µ*m in diameter [8–10]. Peeling off the ILM from the vicinity of a FTMH had dual benefit. Firstly, it eliminated the tangential forces created by glial cells migrating through ILM microrips. Secondly, it induced shearing of the Müller cells' foot plates thereby triggering glial cells proliferation along the interface created by the gas bubble, eventually inducing closure of the hole [11]. While this scenario applied to primary FTMH that were caused by anomalous vitreofoveolar traction, similar success was not achieved in FTMH secondary to trauma [12, 13]. The pathogenic mechanisms entailed in traumatic FTMH formation included direct injury from blunt trauma inducing the classic trampoline effect or from open globe injury, and indirect injury from a propagating shock wave of chorioretinitis sclopetaria or pressure necrosis of the foveal area by subfoveal hemorrhage [14–17]. These mechanisms steered the pathological cascade to a common endpoint, which is hole formation due to tissue loss. This meant posing a significant pathological element that could not be rectified by the aforementioned surgical maneuvers and that hindered anatomical closure and retinal layers' structural recovery. Accordingly, traumatic FTMH acquired notoriety of frequent initial failure, late reopening, and worse final visual outcome compared to the primary variant [18, 19]. The aim of the current study is to compare the efficacy of PPV and ILM peel versus PPV and ILM flap technique (IFT) in terms of anatomic and functional outcomes in patients with traumatic FTMH.

## 2. Patients and Methods

This was a retrospective interventional comparative case series that analyzed data of 40 consecutive patients with traumatic FTMH, who were treated in a private ophthalmic center, Magrabi Eye Hospital, Tanta, Egypt, over the past 5 years. Prior to 2017, our surgical protocol for treating traumatic holes consisted of PPV and ILM peel. As from 2017, all patients underwent PPV and IFT. Preoperative data included age, gender, eye involved, type of trauma, and duration of the disease. Best-corrected visual acuity (BCVA) was measured using the Snellen notation and converted to logarithm of minimum angle of resolution (logMAR) for statistical analysis. Diagnosis of FTMH was based on biomicroscopic examination and optical coherence tomography (OCT) imaging (Cirrus HD-OCT 4000 (Carl Zeiss Meditech, Inc., Dublin, California, USA)) or Heidelberg Spectralis Spectral-Domain OCT (SD-OCT (Heidelberg Engineering, Inc., Heidelberg, Germany)), using high-definition 5-line raster scans and 3-dimensional 512 × 128 macular cube scans passing through the fovea. Biomicroscopically, an FTMH was defined as a central round retinal defect with a rim of elevated retina. Weiss's ring and/or prefoveolar opacity may be present or absent. The size of the central retinal defect was calibrated against the diameter of one of the large tributaries of the central retinal vein close to the optic disc margin [3]. On OCT imaging, an FTMH was defined as an anatomic defect in the fovea involving all neural layers from the ILM to the retinal pigment epithelium (RPE) detected on at least one OCT B-scan. The size of the hole was assessed using the minimum linear diameter (MLD), which was measured using software built-in calipers feature. The MLD was determined by drawing a horizontal line parallel to the RPE between the narrowest hole points in the midretina, i.e., at the shortest distance across the full thickness defect [20]. The study recruited exclusively patients with naïve FTMH with unequivocal history of blunt ocular trauma. Exclusion criteria included recurrent or persistent macular holes following previous surgery, associated retinal detachment or proliferative vitreoretinopathy, significant corneal opacity that would hinder ILM surgery, associated consecutive optic atrophy due to traumatic optic neuropathy, or a follow-up duration less than 3 months. Recruited patients were classified into two treatment arms. Group I included patients who underwent PPV with ILM peel, whereas group II included patients who underwent PPV with IFT. The main outcome measure was closure of the macular hole and restoration of foveal microstructure. Secondary outcome measures were correlation between preoperative MLD, baseline BCVA, duration of the hole prior to surgical intervention, and the anatomical outcome (type of closure and status of foveal microstructure) and the functional outcome (postoperative BCVA), in addition to development of complications. Selection of patients for enrollment in the study and all surgical procedures entailed were undertaken by single vitreoretinal surgeon (HG). The current study was approved by the Institutional Review Board of Magrabi Eye Hospital in Egypt. The study adhered strictly to the tenets of the Declaration of Helsinki (2013 revision). All individuals enrolled in the study received thorough explanation of the surgical procedures entailed, the expected outcomes and possible complications. Afterwards, they were asked to sign an informed consent before undertaking surgery. The consent included a statement that authorized the authors to publish patients' data for research purposes in an anonymous manner that does not allow identification of the patient.

### 2.1. Surgical Technique

Recruited patients who presented with concomitant significant cataract that could hinder adequate visualization during PPV and ILM manipulation underwent PPV combined with standard phacoemulsification with foldable intraocular lens implantation within the capsular bag. Surgical technique in all cases consisted of 23-gauge 3-port PPV, followed by triamcinolone acetonide- (TA-) assisted induction of posterior vitreous detachment (PVD) that was accomplished by applying aspiration over the optic nerve head (ONH) using the vitreous cutter. Once induced, PVD was carried up to the equator. We routinely used TA for ILM peel. After PVD induction, 0.2 ml TA suspension was sprayed onto the macular area. The supernatant suspension is aspirated from the vitreous cavity while allowing large-sized TA particles to settle down over the ILM. Peeling was started by directly pinching the ILM at a point of natural weakness over the inferior temporal arcade using a 23-gauge Eckardt end-gripping forceps (D.O.R.C. Dutch Ophthalmic Research Center (International) B.V., Netherlands). Once a flap is created, it is slightly elevated over the retinal surface then ripped tangentially in a rhexis fashion for at least 2 disc diameters (DD) from the hole. ILM peeling maneuver was performed centripetal to the fovea to avoid enlargement of the hole. During ILM removal, the peeled ILM flap with overlying TA particles was easily identified from the unpeeled ILM. Additional clues for ILM identification included its peculiar glistening reflex, which provided clear contrast with the dull-white appearance of the denuded retina in the peeled area, and/or petechial surface hemorrhages in the peeled area. In cases with inadequate visualization of the ILM, we resorted to ILM-blue stain (D.O.R.C. Dutch Ophthalmic Research Center (International) B.V., Netherlands).

In group I, the ILM was completely removed off the macular hole, whereas in group II, the ILM peel was stopped at the edges of the macular hole forming an island of ILM floating into the vitreous cavity with a 360° attachment to the hole. Redundant peripheral edges of the flap were trimmed by the vitreous cutter using shaving mode with minimal suction. No attempts were made to fold over, dip, or tuck the flap inside the hole to avoid traumatizing the RPE. The retinal periphery was then inspected by scleral depression to check for iatrogenic holes, followed by fluid-air exchange. After removal of the 3 cannulas air/C2F6, hexafluoroethane gas exchange was performed using two 30-gauge needles, one for injection of 14% C2F6 and the other for simultaneous air venting (Supplemental digital content, video 1 demonstrates the IFT using TA). Postoperatively, patients were asked to adopt a drinking-bird positioning protocol, in which the patient had to maintain a face-down posture every other 15 minutes for 50% of his/her waking time for 1 week or until 50% of the gas was absorbed as judged by biomicroscopic examination.

### 2.2. Postoperative Follow-Up

During the postoperative period, patients were examined at 1 day, 1 week, 1 month, and 3 months thereafter. Postoperative data included BCVA, intraocular pressure (IOP) measurement, assessment of macular hole closure biomicroscopically and on OCT examination, and development of postoperative complications.

#### 2.2.1. Macular Hole Closure

On biomicroscopy, macular hole closure was defined as complete apposition of the hole margins and restoration of the foveal light reflex. Patients were then classified according to the closure type and restoration of foveal microstructure on OCT imaging as follows.

*(1) Closure Type*. U-type configuration was defined as closed macular hole with normal foveal contour; V-type configuration was defined as closed macular hole with steep foveal contour, whereas W-type configuration was considered an open flat macular hole with persistent neurosensory retinal defect [21].

*(2) Foveal Microstructure*. Category 1 included eyes with restored external limiting membrane (ELM) and inner segment/outer segment (IS/OS) junction; category 2 included eyes with restored ELM and disrupted IS/OS junction, whereas category 3 included eyes with disrupted both ELM and IS/OS junction. Category 4 included eyes with persistent open hole after surgery.

### 2.3. Statistical Analysis

#### 2.3.1. Independent-Samples *T*-Test

The independent-samples *T*-test procedure compares means for two groups of cases. Ideally, for this test, the subjects should be randomly assigned to two groups, so that any difference in response is due to the treatment and not due to other factors. For each variable, sample size, mean, standard deviation, and standard error of the mean were calculated. For the difference in means, mean and standard error were calculated. The significance of the measured *T*-test value was considered as follows: not significant (NS) when *P* > 0.05, significant (S) when *P* ≤ 0.05 (^*∗*^), highly significant (HS) when *P* < 0.01(^*∗∗*^), and very highly significant (V.H.S.) when *P* < 0.001(^*∗∗∗*^), where *P* is the probability (reflect of null hypothesis).

#### 2.3.2. Analysis of Variance (ANOVA): (F-Test)

ANOVA is a procedure used for testing the differences among the means of two or more treatments. It was noted that if means of subgroups are greatly different, the variance of the combined groups is much larger than the variance of the separate groups. The ANOVA format for the analysis of differences in means is based on this fact.

#### 2.3.3. Correlation Matrix

The bivariate correlation procedure computes Pearson's correlation coefficient that measures how variables are related. Two variables can be perfectly related, but if the relationship is not linear, Pearson's correlation coefficient is not an appropriate statistic. The results of *r* value were checked on *r* table to find out the significant level.

## 3. Results

### 3.1. Baseline Patients' Characteristics

The study included 40 eyes of 40 patients divided in 2 groups. Group I included 28 patients (23 males and 5 females), with a mean age of 21.4 years (range: 5–54 years; SD 13). Mean preoperative MLD was 757 *µ*m (range: 412 *µ*m–1200 *µ*m; SD 221). Mean preoperative BCVA was 1.25 logMAR (Snellen equivalent approximately 20/320) (range: 1.5–0.7 logMAR; SD 0.3). Mean duration prior to surgical intervention was 9 months (range: 0.06–120 months; SD 23.5). Mean duration of follow-up after surgery was 37 months (range: 3–171 months; SD 45). Group II included 12 male patients, with a mean age of 18 years (range: 7–30 years; SD 7). Mean preoperative MLD was 529.5 *µ*m (range: 225 *µ*m-808 *µ*m; SD 148). Mean preoperative BCVA was 1.2 logMAR (Snellen equivalent approximately 20/320) (range: 1.5–0.6 logMAR; SD 0.3). Mean duration prior to surgical intervention was 5 months (range: 0.3–12 months; SD 4.5). Mean duration of follow-up after surgery was 6.3 months (range: 3–12 months; SD 3.3). Statistical analysis revealed no significant difference between both groups in terms of preoperative BCVA and duration prior to surgical intervention, (*P*=0.5), although both groups differed significantly in terms of preoperative MLD and duration of follow-up after surgery, (*P*=0.003  and  0.025, respectively). Baseline patients' characteristics of each group are summarized in Table 1.

### 3.2. Postoperative Anatomical Outcome

#### 3.2.1. Closure Type

**Figure 1:**
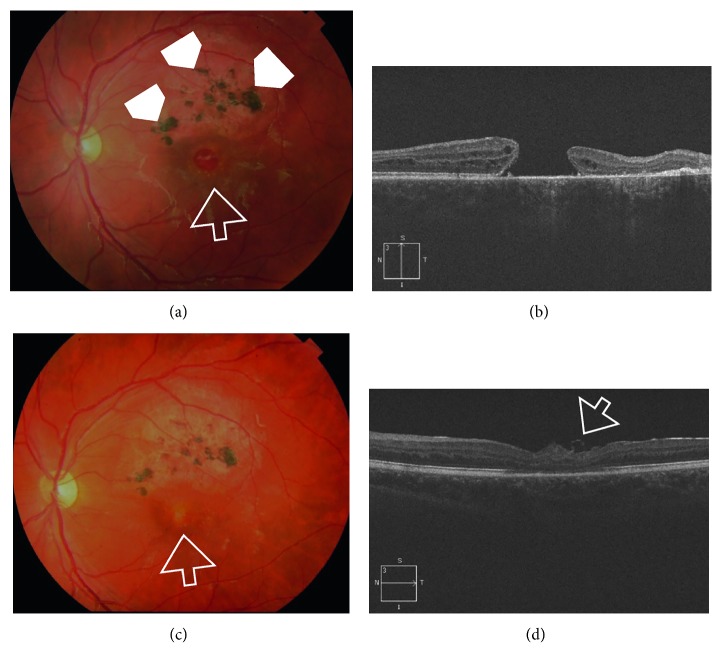
(a) Color photo of the left eye of a 16-year-old male patient. The patient sustained blunt trauma to the left eye with a brick approximately 12 months earlier. His BCVA was 0.05 Snellen. The posterior pole showed a large FTMH approximately 2/3 DD (white arrow). Note the area of diffuse RPE mottling with RPE pigment clumps in the superior vicinity of the hole denoting the chronic course (white arrowheads). (b) High-definition 5-line raster OCT image of the same eye showed large FTMH with MLD 808 *µ*m. Note the cystic thickening at the edges of the hole. (c) Color photo of the same eye approximately 6 weeks after PPV and IFT, showing successful hole closure (white arrow). His final BCVA was 0.2 Snellen. (d) High-definition 5-line raster OCT image postoperatively showing U-type closure. The ELM and IS/OS were not restored. Note the curled edge of the ILM flap (white arrow).

**Figure 2 fig2:**
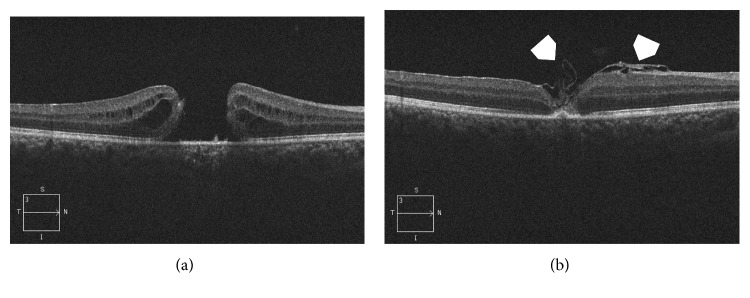
(a) High-definition 5-line raster OCT image of the right eye of a 14-year-old male. The patient was hit by a tennis ball at 1.5 months. The resultant FTMH had MLD 725 *µ*m. His BCVA was 0.05 Snellen. Note the cystic thickening at the edges of the hole. (b) High-definition 5-line raster OCT image of the same eye 2 months after PPV and IFT showing V-type closure. The ELM and IS/OS were not restored. The free ILM flap crumbled into the macular hole (white arrowheads). His final BCVA was 0.4 Snellen.

**Figure 3:**
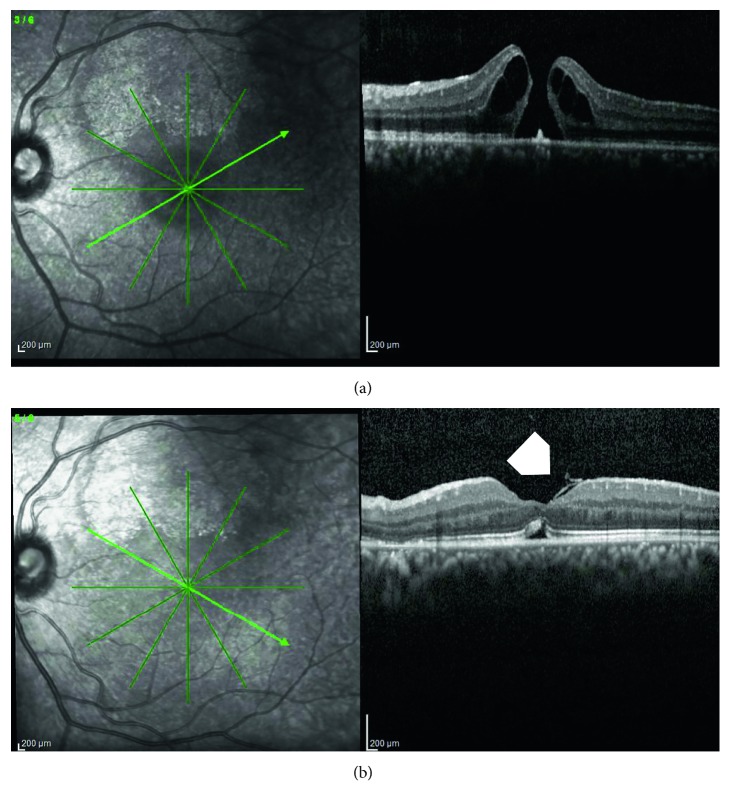
(a) Radial scan OCT image of the left eye of a 16-year-old male. The patient was hit by a closed fist at 15 days. The MLD of the FTMH was 359 *µ*m. His BCVA was 0.1 Snellen. Note the cystic thickening at the edges of the hole with typical pregnant drawbridge appearance. (b) Radial scan OCT of the same eye approximately 2.5 months after PPV and IFT showing U-type closure. Note the residual subfoveal neurosensory detachment, fully restored ELM, and partially restored IS/OS layer. The ILM flap was seen folded on itself into the macular hole (white arrowhead). His final BCVA was 0.4 Snellen.

Postoperatively, V-type configuration was the main closure type detected in group I, as it occurred in 15 eyes (53.5%), W-type configuration occurred in 7 eyes (25%), whereas U-type configuration was detected in 6 eyes (21.4%). In group II, U-type configuration was detected in 6 eyes (50%), V-type configuration was detected in 5 eyes (42%), and W-type configuration was detected in 1 eye (8.3%) (Figures 1 [Fig fig2]–3).

#### 3.2.2. Foveal Microstructure

In group I, 3 eyes (11%) had restored ELM and IS/OS, 5 eyes (18%) had restored ELM and disrupted IS/OS, 13 eyes (46.4%) had disrupted ELM and IS/OS, and 7 eyes (25%) had persistent open holes. In group II, failure of restoration of both layers was detected in 10 eyes (83.3%). Restoration of ELM and IS/OS and persistent open hole were detected in 1 eye (8.3%) each. Therefore, disrupted ELM and IS/OS was the most salient finding in both groups.

### 3.3. Postoperative Functional Outcome

At the end of follow-up, mean BCVA in group I was 1 logMAR (Snellen equivalent 20/200) (range: 1.5–0.2 logMAR; SD 0.4). Mean improvement was 2.5 lines. Three patients (11%) had final BCVA 0.3 logMAR or better (Snellen equivalent ≥ 20/40). On the contrary, mean BCVA in group II was 0.7 logMAR (Snellen equivalent 20/100) (range: 1.3–0.4 logMAR; SD 0.2). Mean improvement was 5 lines. No patient in group II achieved final BCVA 0.3 logMAR or better.

### 3.4. Complications

Cataract formation or progression of incipient cataract that required cataract surgery during the course of follow-up occurred in 12 eyes (43%) in group I and in 3 eyes (25%) in group II. Postoperative anatomical and functional outcomes of each group are summarized in Table 2.

### 3.5. Statistical Correlation of Studied Parameters

#### 3.5.1. Surgical Technique: PPV with ILM Peel versus PPV with IFT

Statistical analysis revealed significant correlation between IFT, better postoperative BCVA, and more favorable closure type of macular hole compared to ILM peel, *p* 0.02 and *p* 0.05, respectively. Failure of restoration of foveal microstructure was the most common outcome of both techniques (64.4% vs. 83.3% in ILM peel and IFT, respectively) (Table 3).

#### 3.5.2. Correlation between Preoperative Parameters and Anatomical Outcome (Type of Macular Hole Closure and Foveal Microstructure)

Statistical analysis in group I and group II, in terms of correlation between preoperative MLD and disease duration prior to surgical intervention versus type of macular hole closure and the degree of restoration of foveal microstructure whether absent, partial, or complete, revealed no statistical significance between these variables.

#### 3.5.3. Correlation between Preoperative Parameters and Final BCVA

Statistical analysis revealed that preoperative BCVA and preoperative MLD were statistically significant parameters influencing the postoperative BCVA in group I patients only, *p* 0.03 and *p* 0.004, respectively. Conversely, duration of disease prior to surgical intervention was not a significant factor influencing postoperative BCVA in both groups (Tables 4 and 5).

## 4. Discussion

In the present study, we report our experience in using PPV and ILM peel versus PPV and IFT for management of traumatic FTMH. Analysis of the anatomical and functional outcomes in group I revealed macular hole closure rate of 75%, of which 21.4% was U-type. BCVA improved by a mean of 2.5 lines. Three patients (11%) had final BCVA 0.3 logMAR or better (Snellen ≥ 20/40). In group II, the macular hole closure rate was 92%, of which 50% was U-type. BCVA improved by a mean of 5 lines. In comparison, Kuhn et al. [22] reported 17 eyes with traumatic macular hole that were treated with PPV and ILM peel. The authors had macular hole closure rate of 100% and improvement of BCVA ≥2 lines in 94% of eyes. It is worthy of note that all eyes in that series had either stage 2 or stage 3 holes at presentation, in comparison to the present study in which the MLD was 757 *µ*m and 529.5 *µ*m in groups I and II, respectively. Johnson et al. [14] studied retrospectively 25 eyes with traumatic macular holes and reported macular hole closure rate of 96% and mean improvement of BCVA ≥ 2 lines in 84% of cases. Twenty-one patients (84%) had stage 2 or 3 holes. ILM was peeled in only 3 cases, and autologous serum was used in 48% of cases. Weichel and Colyer [15] reported macular hole closure rate of 67%. The authors included eyes with both closed and open globe injuries. ILM peel was not done in any case. They did not comment on the size of the macular hole at presentation. A case series by Ou et al. [17] included 5 patients with traumatic macular hole secondary to retinal hemorrhages in shaken baby syndrome. The authors performed PPV and ILM peel for 4 patients and reported macular hole closure rate of 75%. The mean macular hole diameter was 700 *µ*m. A more recent retrospective comparative case series by Ghoraba et al. [23] compared the use of C3F8 and silicone oil in 2 groups of patients who underwent PPV and ILM peel for traumatic macular holes. The authors reported primary closure rate of 81.8% and final closure rate of 90.9% after reoperation, although no information was provided on the preoperative macular hole diameter. The authors mentioned that the overall mean improvement of BCVA was 3 and 4 lines in the silicone oil and C3F8 groups, respectively. There was no information on subgroup stratification in terms of lines of vision gained, lost, or unchanged and how did that correlate with foveal microstructure (Table 6).

To our knowledge, the present study is the first report comparing the outcome of ILM peel technique and IFT in traumatic macular holes. The paucity of literature on outcomes of comparison of both techniques in this particular category of macular holes is a significant deterrent to purposeful validation of our findings in the current study. Moreover, most of the published data on traumatic macular holes were derived from retrospective studies and case reports. Nevertheless, we could compare our results to other studies that compared both techniques in other categories of macular holes that are categorized as recalcitrant macular holes such as large holes and myopic macular holes.  Table 7 summarizes the outcome of different studies that compared PPV and ILM peel versus inverted ILM flap technique in treating different recalcitrant macular holes in comparison with the outcome of the present study.

In summary, the results of the present study demonstrated that IFT is significantly superior to ILM peel in terms of more anatomical macular hole closure and final BCVA. It is worthy of note that, in the IFT group in the present study, we adopted Casini et al. [28] modification of the classic inverted ILM flap technique described by Michalewska et al. [24] in the sense that we did not attempt to invert the ILM flap and fold it inside the hole to avoid damaging the RPE at the bed of the hole. The rationale of our modified approach is that shearing of the foot plates of the Müller cells during ILM peel and residual attachment of the ILM flap to the hole edges would suffice to incite glial cell proliferation and that eventually fills up the macular hole and promotes its closure [11, 24, 31].

Limitations of the current study included its retrospective design that dictated inhomogeneity of the compiled data under the ILM peel group and the IFT group, in terms of number of eyes recruited, macular hole MLD, and duration of follow-up. For instance, 50% of patients in group I had baseline MLD >800 *µ*m versus 8.3% in group II. Given that our statistical analysis revealed significant correlation between preoperative MLD and final BCVA in group I, this could mean that group I patients had worse visual outcome due to initial much larger MLD. However, we could argue that statistical analysis revealed no significant correlation between baseline MLD and macular hole closure or restoration of foveal microstructure in both groups. By extrapolation, the cause of worse final BCVA in group I was related to inferior macular hole closure rate rather than baseline MLD, which adds to the strength of our results as it corroborates the higher efficacy of IFT.

## 5. Conclusion

PPV and IFT is associated with significantly superior anatomic and functional outcomes of traumatic FTMH compared to PPV and ILM peel. Randomized comparative clinical trials focused on surgical management of traumatic FTMH are needed to assert noninferiority or equivalence of PPV and IFT to emerging surgical alternatives before recommending it as a standard surgical approach to traumatic FTMH.

## Figures and Tables

**Table 1:** Baseline patients' characteristics.

Baseline characteristics	Group I *N*=28	Group II *N*=12
*Age (years)*		
Mean	21.4	18
<20	16 (57%)	7 (58%)
20–40	9 (32%)	5 (42%)
>40	3 (11%)	—

*Gender*		
Male	23 (82%)	12 (100%)
Female	5 (18%)	—

*BCVA logMAR (Snellen)*		
Mean	1.25 (∼20/320)	1.2 (20/320)
>1 (<20/200)	19 (68%)	8 (67%)
1–0.7 (20/100–20/200)	9 (32%)	2 (17%)
<0.7 (>20/100)	—	2 (17%)

MLD (*µ*m)		
Mean	757	529.5
<400	—	1 (8.3%)
400–600	9 (32%)	8 (67%)
601–800	5 (18%)	2 (17%)
>800	14 (50%)	1 (8.3%)

*Disease duration (months)*		
Mean	9	5
<1	12 (43%)	2 (17%)
1–6	10 (36%)	6 (50%)
>6–12	2 (7%)	4 (33.3%)
>12	4 (14%)	—

*Follow-up (months)*		
Mean	37	6.3
3–6	11 (39%)	7 (58%)
>6–12	5 (18%)	5 (42%)
>12	12 (43%)	—

BCVA, best-corrected visual acuity; logMAR, logarithm of the minimal angle of resolution; *µ*m, micron; MLD, minimum linear diameter.

**Table 2:** Postoperative anatomical and functional outcomes.

Postoperative outcome	Group I *N*=28	Group II *N*=12
*Closure type*		
U-type	6 (21.4%)	6 (50%)
V-type	15 (53.5%)	5 (42%)
W-type	7 (25%)	1 (8.3%)

*Foveal microstructure*		
Fully restored	3 (11%)	1 (8.3%)
Partially restored	5 (18%)	—
Not restored	13 (46.4%)	10 (83.3%)
Persistent open hole	7 (25%)	1 (8.3%)

*BCVA logMAR (Snellen)*		
Mean	1 (20/200)	0.7 (20/100)
>1 (<20/200)	9 (32%)	1 (8.3%)
1–0.4 (20/50–20/200)	15 (53.5%)	11 (92%)
<0.4 (>20/50)	4 (14%)	—

*Cataract formation*	12 (43%)	3 (25%)

BCVA, best-corrected visual acuity; logMAR, logarithm of the minimal angle of resolution.

**Table 3:** Correlation between surgical techniques (PPV with ILM peel versus PPV with IFT).

Groups	Postoperative BCVA		Foveal microstructure		Closure type	
	Mean	SE	Mean	SE	Mean	SE
Without IFT	0.96	0.07	2.85	0.17	2.03	0.13
With IFT	0.7	0.07	2.9	0.19	1.58	0.19
*T* value	0.19		0.2		1.91	
*P* value	0.02		0.84		0.05	

BCVA, best-corrected visual acuity; IFT, ILM flap technique; ILM, internal limiting membrane; PPV, pars plana vitrectomy; SE, standard error of the mean.

**Table 4:** Group I: statistical correlation between preoperative parameters and postoperative anatomic and functional outcomes.

		Postoperative BCVA (logMAR)	Closure type	Foveal microstructure
Preoperative BCVA (logMAR)	Pearson correlation	0.39^*∗*^	0.12	0.07
	Sig. (2-tailed)	0.039	0.53	0.71
	*N*	28	28	28

Preoperative MLD (*µ*m)	Pearson correlation	0.52^*∗∗*^	0.26	0.28
	Sig. (2-tailed)	0.004	0.17	0.14
	*N*	28	28	28

Disease duration (months)	Pearson correlation	0.12	−0.11	−0.05
	Sig. (2-tailed)	0.53	0.54	0.78
	*N*	28	28	28

BCVA, best-corrected visual acuity; logMAR, logarithm of the minimal angle of resolution; *µ*m, micron; MLD, minimum linear diameter. ^*∗*^Correlation is significant at the 0.05 level (2-tailed). ^*∗∗*^Correlation is significant at the 0.01 level (2-tailed).

**Table 5:** Group II: statistical correlation between preoperative parameters and postoperative anatomic and functional outcomes.

		Postoperative BCVA (logMAR)	Closure type	Foveal microstructure
Preoperative BCVA (logMAR)	Pearson correlation	0.27	−0.17	0.23
	Significance (2-tailed)	0.38	0.57	0.45
	*N*	12	12	12

Preoperative MLD (*µ*m)	Pearson correlation	−0.14	0.19	0.48
	Significance (2-tailed)	0.96	0.54	0.11
	*N*	12	12	12

Disease duration (months)	Pearson correlation	0.09	−0.03	0.07
	Significance (2-tailed)	0.77	0.91	0.81
	*N*	12	12	12

BCVA, best-corrected visual acuity; logMAR, logarithm of the minimal angle of resolution; *µ*m, micron; MLD, minimum linear diameter; *N*, number.

**Table 6:** Review of studies on PPV and ILM peel for traumatic macular hole.

Author	No. of eyes	Surgical technique	Anatomical closure, no. (%)	Functional outcome (mean final BCVA)
Kuhn et al. [22]	17	PPV-ILM peel SF6	17 (100)	6 lines

Johnson et al. [14]	25	PPV-ILM peel (3 cases) C3F8 Autologous serum (12 cases)	24 (96)	≥2 lines in 84% of cases

Ou et al. [17]	4	PPV ILM peel (4 cases) SO, air, C3F8, no tamponade (1 case)	3 (75)	Poor visual outcome

Ghoraba et al. [23]	22	PPV-ILM peel-SO (9 cases) PPV-ILM peel C3F8 (14 cases)	81.8% primary closure, 90.9% after reoperation	3 lines (SO group), 4 lines (C3F8 group)

Current study, 2018 (first comparison between ILM peel technique and IFT)	40	PPV-ILM-C2F6 PPV-IFT-C2F6	75% 92%	2.5 lines 5 lines

BCVA, best-corrected visual acuity; C2F6, hexafluoroethane; C3F8, octafluoropropane; IFT, ILM flap technique; ILM, internal limiting membrane; no., number; PPV, pars plana vitrectomy; SF6, sulfur hexafluoride; SO, silicone oil.

**Table 7:** Review of studies on PPV and ILM peel versus PPV and inverted ILM flap for different types of macular holes.

Author	Macular hole type	Surgical technique	No. of eyes	Anatomical closure (%)	Functional outcome (mean final BCVA)
Michalewska et al. [24]	Idiopathic	PPV, ILM peel, air	51	88%	(Pre-op 0.12)–(post-op 0.17); Snellen
		PPV, inverted ILM flap, air	50	98%	(Pre-op 0.07)–(post-op 0.2); Snellen

Chen et al. [25]	Idiopathic	PPV, inverted ILM flap, C3F8	8	100%	(Pre-op 1.3)–(post-op 0.6); logMAR

Sasaki et al. [26]	Macular hole-associated retinal detachment	PPV, ILM peel	9	55.5%	(Pre-op 1.00)–(post-op 1.02); logMAR
		PPV, inverted ILM flap	6	100%	(Pre-op 1.04)–(post-op 0.6); logMAR
		C3F8 or SF6			

Mete et al. [27]	Myopic	PPV, ILM peel, SF6	36	61%	(Pre-op 0.6) –(post-op 0.58); logMAR
		PPV, inverted ILM flap, SF6	34	94%	(Pre-op 0.7)–(post-op 0.39); logMAR

Casini et al. [28]	Idiopathic	PPV, inverted ILM flap, SF6	41	97.6%	(Pre-op 20/120)–(Post-op 20/30); Snellen
		PPV, modified inverted ILM flap, SF6	40	97.5%	(Pre-op 20/132)–(Post-op 20/35); Snellen

Kannan et al. [29]	Idiopathic	PPV, ILM peel, SF6	30	70%	1.4 lines
		PPV, inverted ILM flap, SF6	30	90%	2.1 lines

Rizzo et al. [30]	Idiopathic, myopic	PPV, ILM peel	300	78.75%	(Pre-op 0.77)–(post-op 0.52); logMAR
		PPV, inverted ILM flap	320	91.93%	(Pre-op 0.74)–(post-op 0.43); logMAR
		C3F8 or SF6			

Current study, 2018	Traumatic	PPV, ILM, C2F6	28	75%	2.5 lines
		PPV, IFT, C2F6	12	92%	5 lines

BCVA, best-corrected visual acuity; C2F6, hexafluoroethane; C3F8, octafluoropropane; IFT, ILM flap technique; ILM, internal limiting membrane; logMAR, logarithm of minimum angle of resolution; no., number; PPV, pars plana vitrectomy; SF6, sulfur hexafluoride.

## Data Availability

The statistical data used to support the findings of this study are included within the article. The data collected from history taking and clinical examination of patients recruited in the current study are confidential. Access to these data is restricted by Magrabi Eye Hospital, Tanta, Egypt, in accordance with the hospital's patients' data protection policy. Data are available for researchers who meet the criteria for access to confidential data through contacting the hospital's medical director Professor Hammouda Ghoraba.

## Additional Points

Traumatic FTMH acquired notoriety of morbid outcome due to sequelae of trauma as tissue loss and retinal atrophy. Despite a myriad of surgical maneuvers, there is no current consensus on the ideal surgical technique. IFT is a promising approach that would improve the final outcome compared to ILM peel.

## Supplementary Materials

Supplementary Materials Supplemental digital content 1. Video demonstrates the IFT using TA.mp4.
